# Genotype by Energy Expenditure Interaction with Metabolic Syndrome Traits: The Portuguese Healthy Family Study

**DOI:** 10.1371/journal.pone.0080417

**Published:** 2013-11-18

**Authors:** Daniel M. V. Santos, Peter T. Katzmarzyk, Vincent P. Diego, Michele C. Souza, Raquel N. Chaves, John Blangero, José A. R. Maia

**Affiliations:** 1 CIFI^2^D, Faculty of Sports, University of Porto, Porto, Portugal; 2 Pennington Biomedical Research Center, Louisiana State University System, Baton Rouge, Louisiana, United States of America; 3 Texas Biomedical Research Institute, San Antonio, Texas, United States of America; Shanghai Institute of Hypertension, China

## Abstract

Moderate-to-high levels of physical activity are established as preventive factors in metabolic syndrome development. However, there is variability in the phenotypic expression of metabolic syndrome under distinct physical activity conditions. In the present study we applied a Genotype X Environment interaction method to examine the presence of GxEE interaction in the phenotypic expression of metabolic syndrome. A total of 958 subjects, from 294 families of *The Portuguese Healthy Family study*, were included in the analysis. Total daily energy expenditure was assessed using a 3 day physical activity diary. Six metabolic syndrome related traits, including waist circumference, systolic blood pressure, glucose, HDL cholesterol, total cholesterol and triglycerides, were measured and adjusted for age and sex. GxEE examination was performed on SOLAR 4.3.1. All metabolic syndrome indicators were significantly heritable. The GxEE interaction model fitted the data better than the polygenic model (p<0.001) for waist circumference, systolic blood pressure, glucose, total cholesterol and triglycerides. For waist circumference, glucose, total cholesterol and triglycerides, the significant GxEE interaction was due to rejection of the variance homogeneity hypothesis. For waist circumference and glucose, GxEE was also significant by the rejection of the genetic correlation hypothesis. The results showed that metabolic syndrome traits expression is significantly influenced by the interaction established between total daily energy expenditure and genotypes. Physical activity may be considered an environmental variable that promotes metabolic differences between individuals that are distinctively active.

## Introduction

The understanding of the wide range of physical activity (PA) and total daily energy expenditure (TDEE) levels in different populations has been of utmost concern in epidemiological research, because of its relationship with health in general [1], and the metabolic syndrome (MetS)—a cluster of interrelated cardiovascular disease risk factors characterized by glucose intolerance, hypertension, dyslipidemia and obesity—in particular [2]. The definition of MetS, and its associated empirical cut-off points of different markers are controversial issues. Moreover, the mechanisms underlying MetS pathophysiology are extremely complex, as it is both genetically and environmentally driven [3]. In the recent Takahata study [4], the results showed that total energy expenditure was, on average, lower among those individuals with the clustering of MetS risk factors, regardless of body mass index status. Also, it has been suggested that moderate-to-high PA levels have a preventive effect on the development of MetS [5], [6]. Further, recent data suggest that distinct PA frequencies and intensities may produce different effects in MetS expression, as a study has showed that everyday activities such as walking and cycling yielded minor effects on MetS whereas high intensity activities performed for more than two hours per week were associated with a lower prevalence of MetS [7].

Phenotypic variation in different MetS indicators at the population level has been studied using different approaches [8], [9], and new efforts have been employed to disentangle the highly complex architecture of their genetic foundations [10]. One aspect that remains to be addressed is the understanding of the differential effects of TDEE and PA on MetS traits via a possible interaction with genetic factors. This possibility was eloquently postulated by Booth and Lee [11] in a revision of Arthur Beaudets concept of environmental-gene interaction [12] in which each “*individual has their own array of disease susceptibility genes that will interact with physical inactivity to produce maladaptive changes in gene-expression that often passes a clinical threshold into a chronic disease phenotype*” (pg. 148). Data supporting this evidence in humans is scarce. Recently, in a 2004 statement by NIH [13] it was indicated that a previously identified type 2 diabetes (T2D) predisposing polymorphism [14] would only express itself concurrently with uncertain genetic susceptibility factors, alongside environmental factors such as PA. In a study on rats, the results showed that after 11 generations of controlled selection in which high aerobic capacity animals were mated, those with high aerobic capacity had 12% lower mean 24 h blood pressure, 16% lower fasting blood glucose, 56% lower fasting plasma insulin, and 63% lower plasma triglycerides ad those with low aerobic capacity [15]. The logical inference was that genetically determined aerobic capacity is related to MetS traits [16].

Contemporary technological advances and new statistical models allow geneticists to study EE and PA and its impact on phenotypic variance in different MetS traits using an escalating wealth of complex genetic models [8]. These developments permit the estimation of complex MetS traits using family data exploiting the elegant flexibility of maximum likelihood estimation techniques [see Elston and colleagues [17]]. These statistical techniques, mostly based on variance components (VC) models, provide a robust framework for statistical inference, which includes parameter and model-likelihood estimation, and associated likelihood ratio testing procedures [18].

Under the VC model approach, we can formally test for genotype x environment (GxE) interaction [19]. Very briefly, this interaction arises when a genotype yields distinct phenotypic expressions under contrasting environments [20]. Here we hypothesize that genotype x EE (GxEE) interaction is a potent determinant of variation in MetS traits. This possibility deserves further consideration, because it can inform the discussion around the preventive and systematic protective effects of EE and PA on MetS traits. For example, in the Nurses Health Study, it was concluded that PA interacts with T2D susceptibility, because among the women whose parents had T2D, a 65% greater risk of developing T2D was found for those who were on the lower quintile of PA level when compared to those in the higher quintile. On the contrary, among the daughters of non-diabetic parents, being in the lower quintile of PA was associated with twice the risk of developing diabetes than being in the most active quintile.

The main purposes of the present study, using a nuclear family design, are to estimate the magnitude of genetic factors responsible for the architecture of MetS traits, and to study potential GxEE interaction.

## Materials and Methods

The Portuguese Healthy Family study, from the Portuguese *Estudo de Famílias Saudáveis Portuguesas* (FAMS), investigates the relationship among MetS traits, PA, physical fitness and body composition in nuclear families. Children and adolescents aged 10 to 18 years were recruited in schools from the north and central regions of mainland Portugal, and were approached to freely participate in the study with their siblings and parents. The ethics committee of the Faculty of Sport, University of Porto, approved the study, and written informed consent, and assent, was obtained from the parents (or guardians). Given that families with 3 or more children are scarce in the Portuguese population [21], a total of 500 families with at least one child were invited to participate in this study. Of these, 294 families agreed to participate with at least two family members (see Table 1).

**Table 1:** Sample descriptive characteristics (means ± standard deviations).

	Fathers	Mothers	Sons	Daughters
	(n = 180)	(n = 253)	(n = 265)	(n = 260)
Age (yrs)	45,36±5,17	43,49±4,47	14,68±2,78	14,40±2,80
TDEE ( kcal/day)	3561.79±962.71	2788.37±527.58	2280.57±774.43	2024.85±568.43
WC (cm)	92,34±10,64	80,98±8,99	72,74±10,41	68,39±8,56
SBP (mmHg)	131,7±14,27	122,12±16,14	117,60±13,06	113,13±10,49
GLU (mg/dl)	97,17±13,19	87,77±13,27	85,39±9,10	83,24±9,01
HDL (mg/dl)	44,58±13,76	55,94±14,37	47,88±13,71	53,30±12,85
TC (mg/dl)	196,72±41,32	181,17±32,17	140,64±24,49	150,44±26,42
TG (mg/dl)	140,33±103,85	109,37±62,63	62,55±30,70	79,57±50,00

Legend: WC – waist circumference; SBP – systolic blood pressure; GLU – glucose; HDL – high density lipoprotein; TC – Total cholesterol; TG – triglycerides.

### Physical Activity

Using a 3 day physical activity diary [22], a trained technician interviewed each subject, recording the dominant activity for each 15-min period during 24 h by using a list of categorized activities. Categories from 1 to 9 refer to increasing levels of energy expenditure (METs) of each activity in which category 1 indicates very low energy expenditure such as sleeping or resting in bed, and category 9 refers to highly demanding physical work such as high-intensity sports. Approximate median energy cost for each of the nine categories in kcal/kg/15 min was used to compute the daily energy expenditure for each individual. The number of 15-min periods for each category was first summed over the 3 day period and weighted by its own median energy cost. Total energy expenditure (TEE) was then calculated by summing over the median energy cost of all nine categories and multiplying by subjectś body weights. Total daily energy expenditure (TDEE (kcal/day)) was then calculated by dividing TEE by 3.

### Blood sampling and measurements of cardiovascular risk factors

Blood samples were collected after an overnight fast of at least 10-12 h. Glucose (GLU), total cholesterol, HDL-cholesterol (HDL), and triglycerides (TG) were analyzed with an LDX point of care analyzer [23]. This method has been previously validated against a laboratory reference method [24], and daily optical equipment checks were made according to manufacturer instructions.

Resting systolic blood pressure (SBP) was measured with an Omron Model M6 (HEM-7001-E) device according to The International Protocol of the European Society of Hypertension [25]. Cuff sizes were modified depending on the size of the participant’s arm. Subjects were seated in an upright position and the right arm sitting on a table at the heart level. The first reading was performed after a 5 minute resting period. The other two readings were performed with three minute breaks in between. The mean of the three blood pressure measurements was used for further analysis. All blood samples and blood pressure analysis were performed between 7:30 am and 10:30 am.

Waist circumference (WC) was measured with a Holtain flexible tape at the level of the smallest waist perimeter, with the subject standing erect with relaxed abdominal muscles and at the end of normal expiration.

### Statistical Analysis

Univariate quantitative genetic procedures as implemented in SOLAR [26] under a special class of the multivariate linear model, namely the variance components (VC) approach, were used to estimate additive genetic and environmental VCs for each of the MS traits. Prior to all modeling, age, age2, sex and their relevant interactions were used as covariates in a preliminary VC model. Residuals were thus derived for each trait and were normalized using an inverse normal transformation, as previously advocated [27], [28]. Heritability estimates (h^2^) were computed using a maximum likelihood approach to estimate variance components under the standard polygenic model as implemented in SOLAR v.4.3.1 software [26].

To test for GxEE interaction, basic initial hypotheses were formulated regarding the variance/covariance relationship of a MetS indicator between family members with different levels of TDEE. As regards GxEE interaction, the fundamental null hypothesis is that the expression of a polygenotype (i.e., aggregate of all genotypes related to the expression of a phenotype) is independent of TDEE level. It can be shown from first principles that if there is no GxEE interaction, the same MetS indicator measured in subjects with different levels of TDEE will have a genetic correlation of 1.0 and the genetic variance will be homogeneous across all levels of TDEE [18], [29]. On the contrary, if GxEE interaction is present, the genetic correlation will be significantly less than 1.0 and/or the genetic variance will not be the same among all levels of TDEE.

The foregoing requires that we model the variance and correlation as functions of TDEE levels. For the genetic variance function (and similarly for the environmental variance function), we modeled the variance using an exponential function to ensure positivity, which is required since any variance is a squared term [18], [29]: 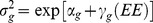, where 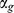 and 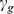 are parameters to be estimated. An additional justification for the exponential function is suggested by the alternative name of this approach, namely the log-linear model of the variance: 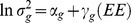. That is, on taking the natural logarithm of the variance modeled as an exponential function, we have the equation of a line. In this form, the variance homogeneity null hypothesis obviously holds for a slope-term equal to 0: 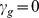. For the genetic correlation function, we modeled the genetic correlation as an exponential decay function of the pairwise differences in TDEE levels: 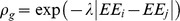, where 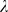 is a parameter to be estimated as a function of the difference in TDEE levels between any two individuals *i* and *j*. Here we also have an elegant reexpression of the interaction null hypothesis, in this case regarding the genetic correlation, in that a genetic correlation equal to 1 is equivalent to 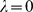. This is because for 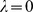, we have 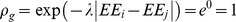.

For reasons detailed in Diego et al. [29], the likelihood ratio test statistics (LRTs) to test 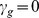 and 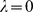 are respectively distributed as 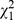, a chi-square random variable with 1 degree of freedom (d.f.), and 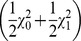, a 50:50 mixture of chi-square random variable with a point-mass at 0, denoted by 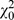, and a chi-square with 1 d.f. Prior to examination of these hypotheses, however, we first confirmed if the overall GxEE interaction model provided a better fit to the data than the standard so-called polygenic model. The LRT for this comparison can be shown to be distributed as 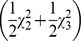 [30].

## Results

Table 1 presents basic descriptive information. Some relatives were not able to fully engage in the data collection procedures. As such, a total of 958 subjects, comprising 180 fathers, 253 mothers, 265 sons and 260 daughters, from 294 families were included. The average family size was 3.3 subjects. Families are, on average, young and the results are as expected as the mean values for all the MetS traits were consistently higher in parents than in offspring. Also, with the exception of HDL cholesterol, MetS indicators were higher in fathers than in mothers. The MetS profiles of sons and daughters were similar with daughters showing higher mean levels of TC, HDL and TG.

All MetS indicators showed highly significant h^2^ estimates ranging from 0.21 (TG) to 0.59 (HDL) (Table 2), meaning that there are strong additive genetic factors affecting their expression in family members that may justify a further specific analysis of their genetic architecture.

**Table 2:** Heritability estimates (h^2^) and corresponding 95% confidence intervals (95%CI) of the different phenotypes in the Portuguese Healthy Families Study

Trait	h^2^	Std. Error	p-value	95%CI
WC (cm)	0,34	0,07	<0,001	0,22–0,45
SBP (mmHg)	0,40	0,07	<0,001	0,27–0,51
GLU (mg/dl)	0,29	0,07	<0,001	0,18–0,40
HDL (mg/dl)	0,59	0,06	<0,001	0,48–0,69
TC (mg/dl)	0,51	0,07	<0,001	0,39–0,62
TG (mg/dl)	0,21	0,08	0,002	0,09–0,33

Legend: WC – waist circumference; SBP – systolic blood pressure; HDL – high density lipoprotein; TC – Total cholesterol; TG – triglycerides.

To test for the influence of TDEE and PA on the expression of MS indicators, the polygenic model was compared to the GxEE model by means of a log-likelihood ratio test (see Table 3). The GxEE interaction model is significantly better than the polygenic model for WC, SBP, GLU, TC and TG, meaning that the GxEE interaction model fits the data better than the polygenic model for these five traits.

**Table 3:** Results of log-likelihood ratio tests (LRT) and respective p-values contrasting a polygenic model vs a GEE model for each of the MS indicators.

Trait	Polygenic LnL	GxPA LnL	LRT	p-value
WC	–380,061	–319,731	120,660	<0,0001
SBP	–370,926	–364,625	12,601	0,004
GLU	–444,331	–384,913	118,835	<0,0001
HDL	–340,745	–340,542	0,408	0,877
TC	–357,813	–343,478	28,669	<0,0001
TG	–380,194	–331,080	98,228	<0,0001

Legend: WC – waist circumference; SBP – systolic blood pressure; HDL – high density lipoprotein; TC – Total cholesterol; TG – triglycerides; LnL – log-likelihoods; LRT – Likelihood ratio test.

However, to verify if there is GxEE interaction, the full model was compared to its constrained alternatives (i.e. setting 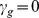
or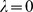) for WC, SBP, GLU, TC and TG.

**Figure 1:**
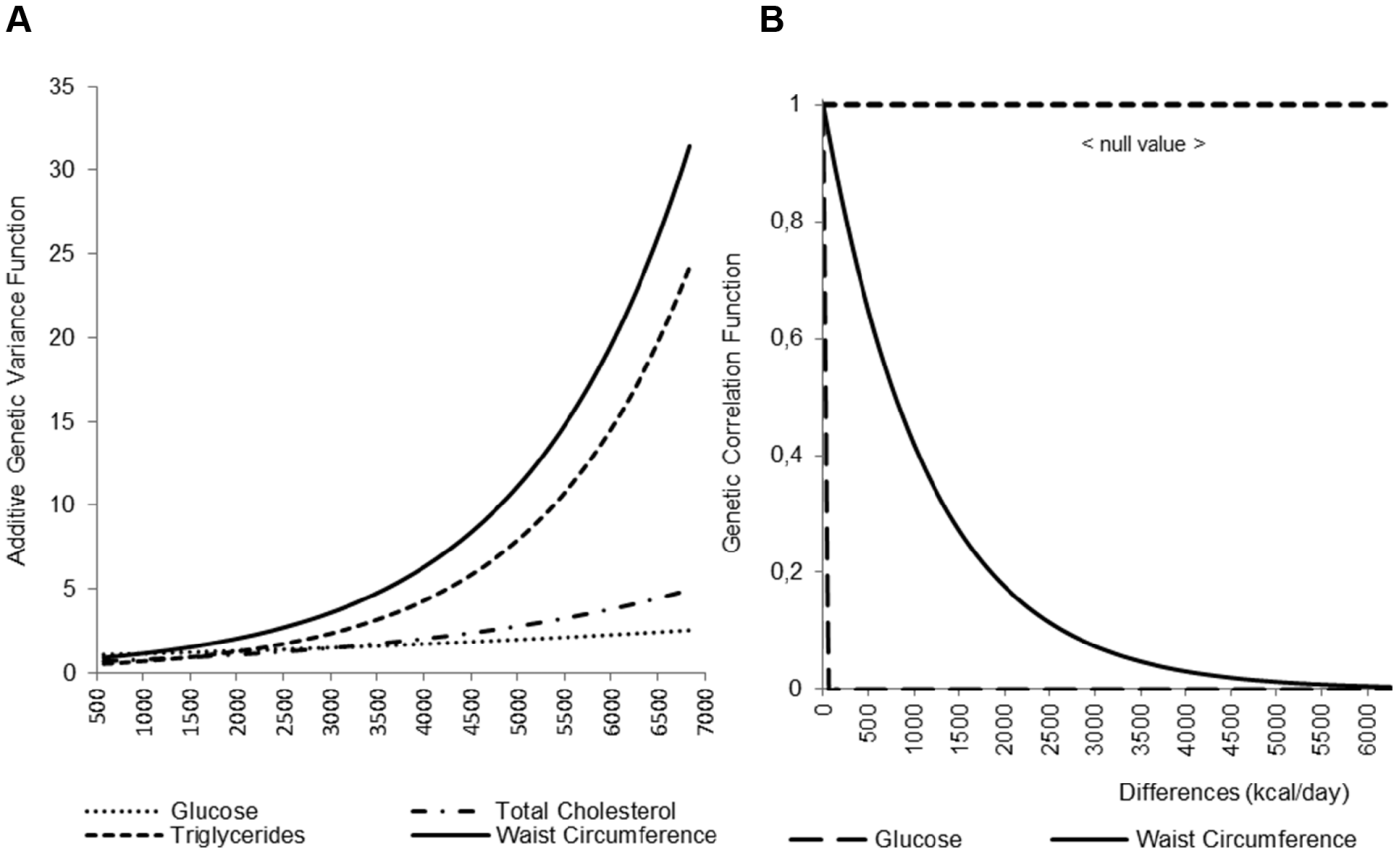
Genotype X Total Daily Energy Expenditure genetic variance (a) and Genotype X Total Daily Energy Expenditure genetic correlation (b).

In Figure 1, we display the results for those traits that showed significant variance heterogeneity and a correlation function that is significantly different from 1. For WC, GLU, TC and TG, significant GxEE interaction was due to rejection of the variance homogeneity hypothesis; i.e., variance heterogeneity. For WC and GLU, the null hypothesis that the genetic correlation (ρG) equals 1 was also significantly rejected. Figure 1a highlights that, for GLU, TC, TG, and WC, genetic variance increases with increasing levels of TDEE. On the other hand, figure 1b demonstrates that, for GLU and WC, the genetic correlation decreases as the differences between TDEE level increases among family members. It may be noticed that genetic correlation for GLU decays to 0 almost instantaneously. However, this is simply an artifact of the two correlations being plotted on one scale and therefore the genetic correlation that decreases at a faster rate seems to immediately go to 0. This is clarified in the next figure.

**Figure 2:**
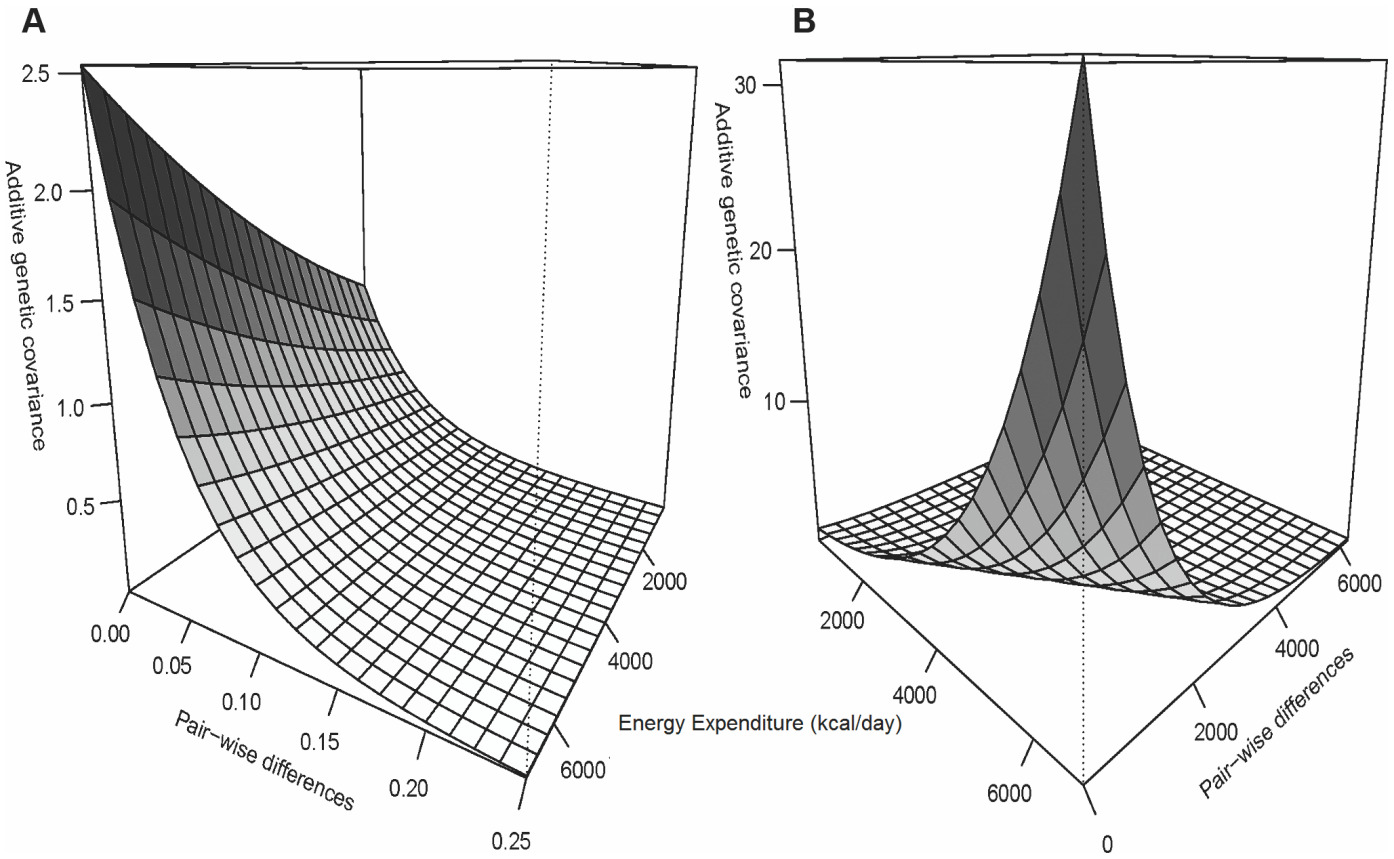
Genetic covariance function for Fasting Glucose (a) and Waist Circumference (b). Total Daily Energy Expenditure units are in kcal/day.

Figure 2 illustrates that, for GLU and WC, GxEE interaction is a joint function of genetic variance heterogeneity and a genetic correlation function not equal to one. Thus, we express them jointly as a covariance function in the vertical axis. Due to space restrictions on the 3-dimensional plot, we abbreviated the corresponding axes in figure 1 to TDEE and pair-wise differences. Moreover, because the traits change at different rates, and to show the correlation functions for both traits at the appropriate resolution, the pair-wise differences are shown on different scales. In fact, the GLU correlation function curve is decreasing at a much faster rate than WC, forcing us to change the scale.

## Discussion

The present report aimed to assess the genetic variance present in MetS traits as well as to examine potential GxPA interaction that has an effect on MetS traits. Our results confirm the importance of genetics on MetS traits with all h^2^ being significant and, more interestingly, highlight the importance of GxEE interaction in the phenotypic determination of MetS traits.

To the best of our knowledge this is the first attempt to apply a GxE interaction analysis to better understand the differential relationship between TDEE and PA with MetS risk factors. A GxE interaction effect is present when the phenotypic expressions of an environmental factor or behavior is conditional to the genotype of an individual. This study provides evidence that there is a genetic basis for the variability in quantitative measures of the metabolic syndrome that is mediated by energy expenditure and/or physical activity.

The present findings confirm that MetS traits are highly heritable in agreement with previous results [31], [32]. HDL (h^2^ = 0.59) was the most heritable of the MetS traits which is consistent with results from elsewhere ranging from 0.46 in Tehran Lipid and Glucose Study (TLGS), and 0.63 from the Family Heart Study (FHS) population. On the other hand, TG (h^2^ = 0.21), in disagreement with the data from TLGS (h^2^ = 0.36) and FHS (h^2^ = 0.48) [31], [33], was the least heritable trait. These differences could be attributable to distinct genetic architectures, namely gene frequencies and their value, as well as distinct environmental factors specific to each population. Also, distinct analysis strategies (e.g., Tang and colleagues [31]) made adjustments for a set of confounders that were not used in the present report], and different sample sizes might explain some of the variability in the TG heritability estimates.

It is well established that PA has a preventive effect on a variety of morbidities associated with cardiovascular diseases [9]. For instance, studies on familial hypercholesterolemia (FH) in Utah families seem to demonstrate this very point. While FH, which leads to early-onset coronary heart disease, is caused by mutations in the LDL-receptor gene (LDLR), it was shown that heterozygous carriers of the LDLR mutation lived into their eighth and ninth decades in the 19th century but their descendants in the 20th century, who were also heterozygous carriers, lived only into their third and fourth decades [34], [35]. These investigators documented that the carriers who lived in the 19th century were relatively more physically active and enjoyed a more nutritious diet than the carriers who lived in the 20th century. However, it has been shown that there is a considerable heterogeneity in the response to PA leading to distinct signals in terms of cardiovascular risk factors [36]. For example, in the HERITAGE Family Study, after 20 weeks of supervised training sessions, a significant mean 3.6% increase in plasma HDL was observed together with a high inter-variability in responsiveness to training, ranging from a mean 9.3% decrease in Quartile 1 of HDL-C response to a mean 18% increase in Quartile 4. Moreover, the authors verified that only 15.5% of the variability was due to baseline variables and training adaptations, concluding that only a minor extent of the adaptation could be predictable by nongenetic factors. This complex and controversial theme has been addressed in a recent paper by Bouchard et al. [37] in which some participants, when exposed to regular exercise, ended up having worsened metabolic profiles. The preceding raises questions related to the importance of genetic susceptibility in explaining the variability in the response to similar levels of PA.

Our results showed that there is additive genetic variance heterogeneity for GLU, TC, TG, and WC across TDEE levels, and that for WC and GLU, the genetic correlation between distinct levels of TDEE was different from 1. These results may be related to work demonstrating that physical activity and inactivity have anti- and pro-inflammatory effects, respectively [38], [39]. It is widely believed that the MetS and MetS-associated diseases such as type 2 diabetes (T2D) and cardiovascular disease (CVD), are caused in a large part by chronic sub-clinical inflammation [40], [41]. That we observed increasing additive genetic variance heterogeneity for GLU, TC, TG, and WC with increasing TDEE levels is consistent with upregulation in the genes involved in anti-inflammatory processes. Recent work [42] showed significant SNP–moderate to vigorous PA interactions on BMI-for-age Z-scores in European-American at *GNPDA2* and *FTO* genes, and in Hispanic-American at *LZTR2/SEC16B*. In 2011, a robust meta-analysis of 218,166 adults and 19,268 children found that, in adults, PA attenuated the effect (p-value for interaction  =  0.001) of the minor (A2) allele of *rs9939609* on obesity. Even though this interaction failed to be statistically significant in children and adolescents, in adults the minor allele of *rs9939609* increased the odds of being obese less in the physically active group (odds ratio  =  1.22/allele, 95% CI 1.19–1.25) than in the inactive group (odds ratio  =  1.30/allele, 95% CI 1.24–1.36). These results seem to suggest that there are genes associated with increased MetS risk factors, such as obesity, that actually interact with different EE levels. Further, that we observed a genetic correlation function different from 1 across TDEE differences is consistent with the activation of genes involved in pro- and anti-inflammatory processes at relatively low and high PA levels, respectively [43].

We speculate that potential epigenetic changes driven by EE might be linked to a major epigenetic modification - DNA methylation - that suppresses gene expression by modulating the access of the transcription machinery to the chromatin or by recruiting methyl binding proteins [44]. In a recent paper by Barrés et al. [45] global methylation values decreased after intense exercise, even after controlling for the effect of hemoglobin mRNA content. More specifically, the results highlighted that captured methylated promoters for metabolic genes, previously linked to type 2 diabetes [46], were lower after acute exercise, leading the authors to suggest that acute exercise induces gene-specific DNA hypomethylation in human skeletal muscle [45].

As mentioned earlier we were unable to find similar papers to which we could compare our results and assess the suitability of our interpretations. It is quite interesting that despite the great wealth of data on PA and MetS, be it from descriptive and/or interactions studies, results are still lacking on the complex and divergent effects of PA on MetS traits given individualś genotypes. Perhaps this may be due to the complex nature of MetS, the statistical challenges that GxE interaction poses, and the necessity of having large samples of families. Moreover, with the development of DNA sequence analysis, and the possibility of running genome wide associations scans (GWAs), many investigators have devoted their attention to finding specific loci associated with the phenotypic variability of MetS traits [47]. We feel as though it is extremely important to be able to merge the new evidence that are now being brought to life by GWAs with the results that are shown in this report and that consubstantiate that there is an underlying effect of genetic susceptibility in phenotypic expression of MetS traits.

Notwithstanding the importance of the present results, the fact that this study is based on a free health check-up, that may not be representative of the general Portuguese population, is a limitation as well as the relatively young and healthy sample of families may limit the generalizability of the results to older individuals who are more prone to develop MetS. Also, the lack of information about resting metabolic rate may influence the PAEE results, although previous work [46], [48] with this 3-day diary never considered this possibility. Another limitation is related to a lack of information on nutritional habits, although this information is never easy to evaluate. However, the large sample size, the reliance on continuous data for MetS traits, the use of state of the art statistical procedures and the novelty of the analysis in TDEE and PA genetic epidemiology research, are strengths of the present study.

In conclusion, the present results demonstrate that MetS trait expression is significantly influenced by the way in which the genotype “deals” with distinct TDEE levels. As such, PA may be considered an environmental variable that promotes metabolic differences between individuals that are distinctively active. This is valuable information for health practitioners. More efforts should be devoted to identify specific loci that control MetS traits and to test if those loci are regulated or not by TDEE and/or PA.
